# Tensile Fracture Behavior and Failure Mechanism of Additively-Manufactured AISI 4140 Low Alloy Steel by Laser Engineered Net Shaping

**DOI:** 10.3390/ma10111283

**Published:** 2017-11-09

**Authors:** Hoyeol Kim, Zhichao Liu, Weilong Cong, Hong-Chao Zhang

**Affiliations:** 1 Department of Industrial, Manufacturing, and Systems Engineering, Texas Tech University, Lubbock, TX 79409, USA; zhichao.liu@ttu.edu (Z.L.); weilong.cong@ttu.edu (W.C.); hong-chao.zhang@ttu.edu (H.-C.Z.); 2 School of Mechanical Engineering, Dalian University of Technology, Dalian 116023, China

**Keywords:** fractography, tensile test, lack-of-fusion defects, carbides precipitation, oxide formation, laser engineered net shaping

## Abstract

AISI 4140 powder was directly deposited on AISI 4140 wrought substrate using laser engineered net shaping (LENS) to investigate the compatibility of a LENS-deposited part with the substrate. Tensile testing at room temperature was performed to evaluate the interface bond performance and fracture behavior of the test specimens. All the samples failed within the as-deposited zone, indicating that the interfacial bond is stronger than the interlayer bond inside the deposit. The fracture surfaces were analyzed using scanning electron microscopy (SEM) and energy disperse X-ray spectrometry (EDS). Results show that the tensile fracture failure of the as-deposited part is primarily affected by lack-of-fusion defects, carbide precipitation, and oxide particles inclusions, which causes premature failure of the deposit by deteriorating the mechanical properties and structural integrity.

## 1. Introduction

AISI 4140 is one of the representative medium carbon and low alloy steels and widely used for manufacturing of many industrial components, such as gears, shafts, and rotors, due to its good hardenability, strength, toughness, and wear resistance [1,2,3,4]. However, when the components made of this alloy steel are exposed to harsh operating conditions, such as surface rolling and sliding contact, during their service life, they are susceptible to serious surface damage, such as micropitting, abrasive wear, and corrosion, which could accelerate premature failure and shorten the life cycle of these critical and expensive components [5,6]. Thus, it is essential to restore the worn-out or damaged components so as to lengthen their service life [7]. 

Traditionally, once the failure occurs, the damaged component is discarded and replaced, resulting in excessive material waste and loss of high value-added components [8,9]. Taking into consideration high costs and long lead time for manufacturing of a new component, effective surface repair technology is highly required [10]. Moreover, there also exists an increased industrial demand for all the components with higher performance and durability [11]. Therefore, it is imperative to repair and reconstruct the worn and damaged components in order to extend the life span and minimize waste of expensive materials, economic losses, downtime, and embodied energy, thereby increasing the industrial competitiveness [12,13].

To prevent these surface failures from the external working environments, surface treatment processes, such as carburizing [11], boriding [14], nitriding [15], thermal spraying [16], and high-velocity oxygen fuel (HVOF) [17], have been commonly applied in practice to improve the surface integrity and properties, such as surface hardness, wear, and oxidation resistance. However, these conventional processes are not generally appropriate for repair or coating due to the difficulty of thickness control, high thermal stress, large heat-affected zone (HAZ), and weak bonding strength [18].

Recently, additive manufacturing (AM) technologies are increasingly used through different approaches, such as selective laser melting (SLM), electron beam melting (EBM), and laser engineered net shaping (LENS), to fabricate various solid and complex metallic materials, such as steels, bronze, and titanium [19,20,21,22,23]. LENS, also known as direct laser deposition, is a laser-based additive manufacturing process that uses a high power laser as a heat source to create a melt pool on the surface of a solid substrate and melt powders through powder feeding nozzles to build a near net shaping without serious post-processing. LENS has been extending its application to the surface treatment area and has received a great deal of attention as a promising technique to improve surface properties, such as wear and corrosion resistance. The surface is considered as the most important part of engineered components since it is exposed to wear and corrosion environments [24]. It can also offer protective coatings and repairing for the critical surfaces of industrial components due to its additive nature with minimum post process finishing. These coatings have shown excellent metallurgical bonding qualities with a smaller HAZ and also the interface bond between the coating and substrate is much stronger compared to conventional surface treatment processes [25]. Thus, the parts coated or repaired by LENS not only prolong the lifetime of the components subjected to severe working conditions, but also reduce costs and downtime.

Interface bonding between the laser-repaired part and the original base substrate plays a vital role in determining the overall performance of the final component since weak interfacial adhesion will bring about fatal failure caused by detachment of the coating from the base or cracking along the interface [10]. Only when the interface is durable, the repaired part will be functional [26]. Therefore, it is crucial to join two materials with a good interface bond to ensure integrity and reliability of the whole part. 

To date, some studies of AISI 4140 alloy steel processed by LENS have been conducted to investigate its microstructure and mechanical properties [27,28]. However, only a limited number of investigations on LENS-based direct joining between metallic powder and corresponding wrought substrate have been carried out so far and, additionally, they were primarily limited to nickel-based Inconel 718 [29,30]. To the best of our knowledge, no investigation on LENS-deposited AISI 4140 powder on the corresponding substrate has been reported in the literature. Particularly, there is a lack of knowledge on the interface bonding and fracture behavior of a hybrid part which is composed of half-additive part and half-subtractive substrate. Most of surface treatment research have mainly concentrated on wear and hardness properties owing to a thin deposition layer. When an area to be repaired is wider and deeper, thick multilayered deposition is indispensable.

Therefore, this study aims to investigate the compatibility of LENS-deposited AISI 4140 powder with its wrought counterpart by focusing on the interface bonding and fracture behavior of the hybrid fabricated specimens. To do so, a tensile test is performed to examine the interface bond and fracture behavior of the specimens. To reveal the fracture failure mechanism, the fracture surface is characterized by scanning electron microscopy (SEM) with energy disperse spectrometry (EDS).

## 2. Materials and Methods

AISI 4140 steel (McMaster-Carr Co., Elmhurst, IL, USA) round bar 9 mm in diameter and 30 mm length was used as a substrate, and its typical chemical composition (wt %) is C 0.38–0.44, Si 0.20–0.35, Mn 0.75–1.0, Cr 0.8–1.1, Mo 0.15–0.25, S ≤ 0.04, P ≤ 0.035, and Fe as the balance. The substrate surface was first machined and ground on abrasive silicon carbide papers to eliminate oxidized layers and then degreased with acetone before LENS deposition. Commercially-available prealloyed gas-atomized (GA) AISI 4140 powder was utilized in as-received condition with the particle size range of 44−105 µm, and its nominal composition (wt %) is C 0.44, Si 0.21, Mn 0.90, Cr 1.0, Mo 0.21, S ≤ 0.02, P ≤ 0.01, and Fe as the balance.

**Figure 1:**
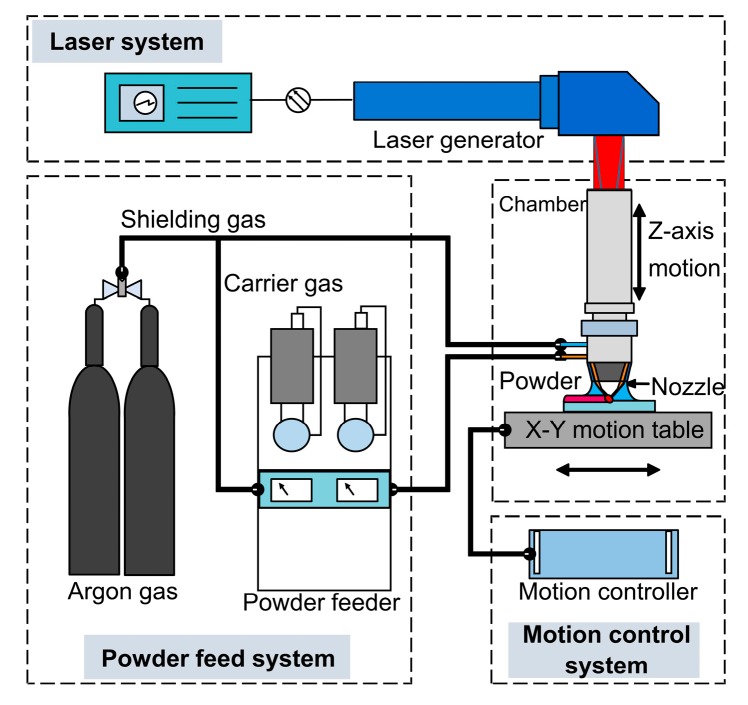
Illustration of the LENS^TM^ system employed in the experiment.

The fabrication process was undertaken using a LENS^TM^ 450 machine (Optomec Inc., Albuquerque, NM, USA) that consists of a 400 W IPG fiber laser system, a pneumatic powder feeder system, and a computer-controlled motion system. The powder is delivered by argon carrier gas from the powder hoppers and injected on a substrate by four jet deposition nozzles. The powder flow rate is adjusted with revolutions per minute (rpm) and controlled by the powder feeder system equipped with rotational motors. Highly-focused laser power is used to melt powder particles and create a molten pool, and the motion control table is moved according to preset travel paths to fabricate an object in a layer by layer style. Figure 1 illustrates a schematic diagram of the LENS^TM^ system employed in the experiment.

**Figure 2:**
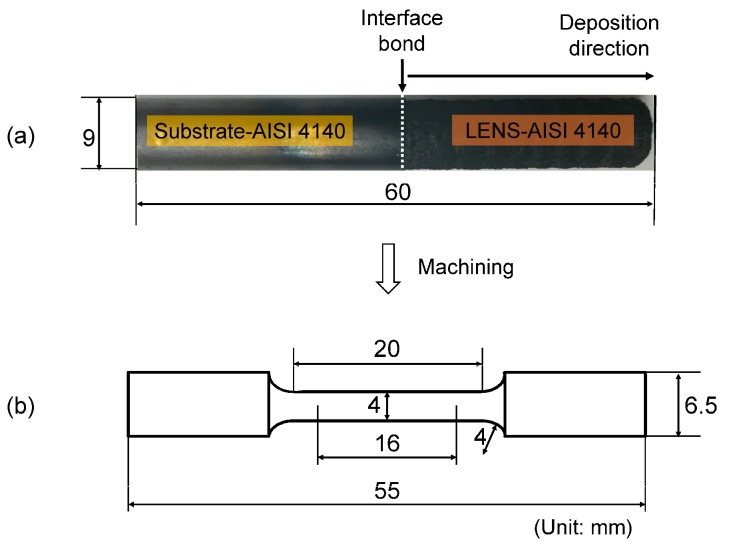
(**a**) Cylinder-shaped pillar after LENS deposition and (**b**) the dimensions of the hybrid test specimen after machining.

A series of preliminary investigations were conducted prior to fabricating the final specimen to obtain a stable geometrical shape and good adhesion to the substrate. The following process parameters were selected: laser power, 380 W; powder feed rate, 2 rpm; travel velocity, 8.47 mm/s; hatch angle, 60°; hatch spacing, 0.76 mm; carrier gas flow rate, 6 L/min; and layer thickness, 0.43 mm. A cylindrical pillar from AISI 4140 powder with the same dimensions as the substrate was directly deposited on top of the substrate surface (Figure 2a). The density of the LENS-processed samples was measured using Archimedes’ principle. The average relative density of the samples was 94.8 ± 3.5%. The fabricated part was machined to the final test specimen dimensions (Figure 2b). A total of three specimens were fabricated under the same conditions with the same size, and tensile tests at room temperature were conducted with 4 mm diameter and 16 mm gauge length specimens using an Instron MTS universal testing machine (Instron Corp., Norwood, MA, USA). Fractography was performed using Zeiss Crossbeam 540 SEM (Carl Zeiss AG, Oberkochen, Germany) workstation equipped with EDS to analyze fracture surfaces and to quantitatively detect elemental composition and identify phase formation.

## 3. Results and Discussion

### 3.1. Interface Bond and Tensile Fracture Behavior

Particular attention was paid to interface bonding between the LENS-deposit and the substrate counterpart since a good interface bond is a prerequisite for compatibility of the as-deposited metal with the substrate and structural integrity of the whole part [10]. Another matter of concern is that mechanical properties of this hybrid structure are required to be similar to those of the original base material [31]. Hence, its interfacial bond and fracture behaviors were evaluated by means of a tensile test.

**Figure 3:**
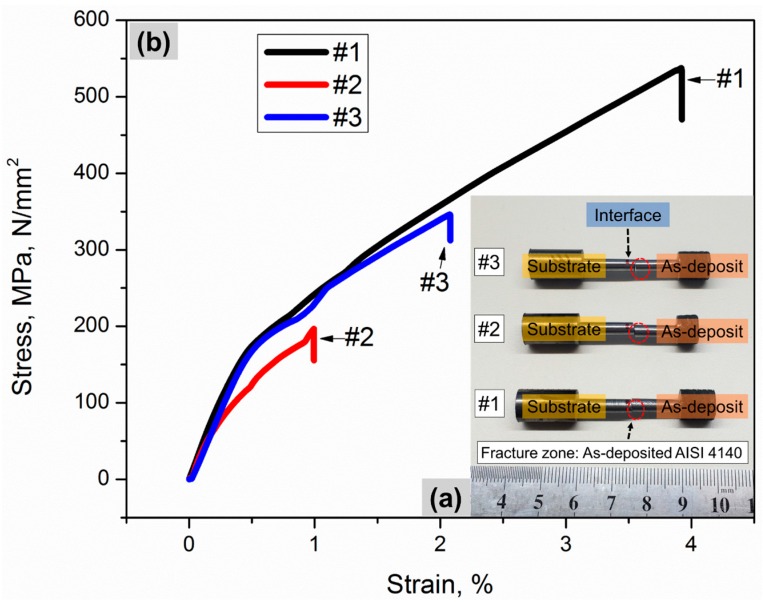
(**a**) Hybrid specimens that fractured at as-deposited zone indicated by dotted circles after tensile testing and (**b**) the corresponding stress-strain curves.

Figure 3 shows each fractured specimen after the room-temperature tensile test and a corresponding stress-strain curve. The substrate accounts for the left side of the specimen, and the as-deposited AISI 4140 occupies the right side of it. As seen in Figure 3a, all the specimens fractured at the as-deposited region. No indication of fracture failure was observed at the substrate and the interface, which implies that the substrate and the interface is stronger than the as-deposited part in the hybrid specimen. Therefore, it can be deduced that the strength of the interface bond between the as-deposited part and the substrate is higher than that of the interlayer bond within the as-deposited section.

The tensile properties of the hybrid specimens were compared with those of AISI 4140 wrought in terms of ultimate tensile strength (UTS), yield stress (YS), and plastic elongation in Table 1. It can be said that the tensile properties of the samples represent those of the as-deposited AISI 4140 because all the fractures took place at the deposited zone. The average UTS, YS, and elongation values of the hybrid samples (360, 237 MPa, and 2.3%, respectively) were lower than those of the wrought counterpart (720, 655 MPa, and 4%, respectively). 

As observed in Figure 3b, the tensile behaviors were highly varied among the specimens, which is attributed to the metallurgical defects, such as partially-melted powder and lack-of-bonding porosity [33,34]. No prominent necking behavior was presented from the fractured AISI 4140 parts (Figure 3a). Furthermore, the cross-sectional morphology of the fractured specimens indicated flat and smooth surfaces, indicating a brittle fracture mode.

### 3.2. Fracture Surface and Failure Mechanism

From the results of the tensile test, all the samples fractured within the laser-deposited AISI 4140 region instead of the interface or the substrate, indicating that the interlayer bonding is weaker than the interface bonding since tensile fracture normally takes place at the weakest location of the specimen. To investigate the major causes that make the as-deposited part weak, the fracture surfaces were examined by SEM and EDS.

#### 3.2.1. Fracture Morphology and Defects

**Figure 4:**
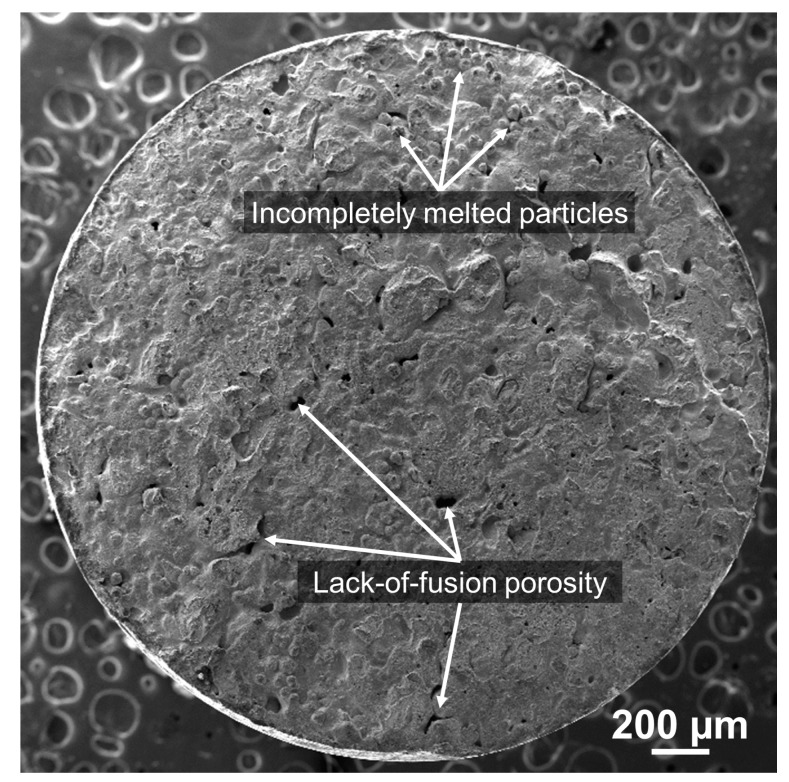
Overall fracture morphology of specimen #3 with insufficiently melted powder particles and porosity.

In Figure 4, the overall fracture surface of specimen #3 with the intermediate tensile properties (Figure 3b) predominantly presented a smooth brittle fracture mode. During the tensile test, the brittle fracture occurred suddenly with little plastic deformation. Additionally, the fracture angle between the applied tensile loading axis and the fracture surface was nearly perpendicular and exhibited a relatively flat fracture surface, indicating that the crack propagates parallel to the macroscale plane of the maximum normal stress by continuously breaking the atomic bonds along specific cleavage planes [35].

A cluster of spherical particles was largely distributed on the fracture surface as marked in Figure 4, which was regarded as partially melted powder during LENS process. As a result, voids were formed between the unmelted or partially-melted powder particles due to excessively blown powder into the molten pool [29], misalignment of the deposition head [36,37], or insufficient energy density [38,39].

Moreover, relatively larger and irregular pores were clearly seen throughout the fracture surface owing to a localized lack of fusion during deposition, which resulted in the existence of gaps between deposited tracks as indicated by arrows in Figure 4. This is most likely attributable to locally insufficient energy density absorbed by the powder particles since porosity has an inverse relationship with absorbed energy per unit length of a deposited track [36,40].

The main downside of direct laser deposition processes are process-induced defects, such as unmelted particles and lack-of-fusion porosity. It is known that they can significantly reduce the mechanical properties such as elastic modulus, ductility, microhardness, and bonding strength of the laser-deposited part by leading to a drop in stress-strain tensile curves, early deviation from the linear elastic response at low tensile stress loading, as well as premature fracture failure [34,41,42,43] due to reduced density and weakened interlayer bond strength of the as-built part [44,45]. Therefore, the tensile test result showed highly-deviated tensile behaviors between the test specimens, as seen in Figure 3b, and the tensile properties of the as-deposited part were remarkably lower than those of the wrought AISI 4140 (Table 1).

**Figure 5:**
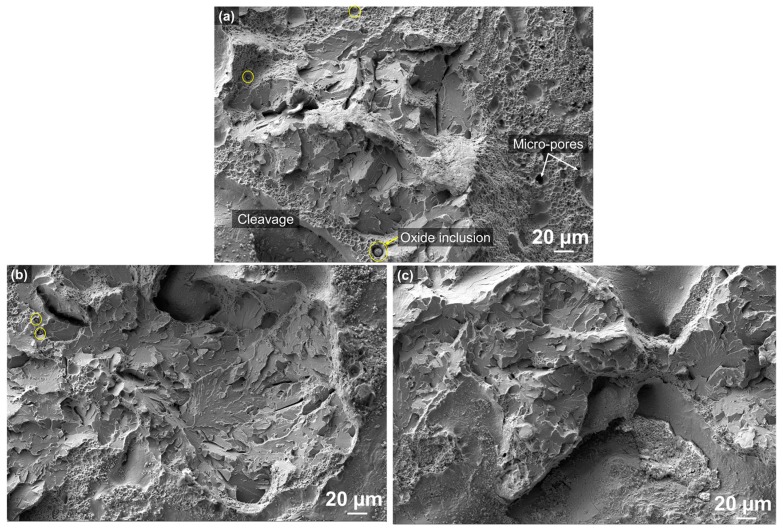
(**a**–**c**) High magnification views of the fracture surface after tensile testing showing a transgranular quasi-cleavage fracture mode.

From the high-magnification views on the fracture surface, more details of fracture morphology and defects were revealed, as seen in Figure 5a–c. The brittle cleavage was predominant with a very small amount of a localized dimpled zone, which shows a quasi-cleavage fracture mode (Figure 5a). The locally-dimpled microvoids that coalescence represent a ductile fracture, whereas the tearing cleavage deformation indicates a brittle fracture, which is commonly found in steels as observed in other studies [46,47,48,49,50]. The microscopic mechanism of the cleavage fracture is specified as follows: (i) crack nucleation at cementite particle; (ii) propagation of the microcrack nucleus across the particle/matrix interface along a cleavage plane of the neighboring grain; and (iii) propagation of the grain-sized crack to neighboring grains across the grain boundaries leading to final failure [35,51,52]. The cleavage-type morphology mostly appeared to be smooth and flat, suggesting a lack of bonding between adjoining layers or tracks. 

Spherical-shaped micro-pores were also identified in the dimpled rupture zone as indicated by arrows in Figure 5a, which was on account of the entrapped gas inside the gas-atomized (GA) hollow powders [30,36,37,53,54]. The trapped gas inside the powders could not come out of the melt pool because of the fast cooling nature caused by the LENS process, which ended up forming the micropores in the as-deposited part. Furthermore, spherical oxide inclusions were observed in the dimple fracture area as indicated by circles in Figure 5a. The details on chemical element analysis using EDS for the inclusions will be discussed in Section 3.2.3.

Figure 5b,c also represent the typical transgranular crack morphology with the river patterns which indicate the direction of localized crack propagation [45,55,56]. It was observed that the presence of this transgranular cleavage fracture mechanism in steel is related to iron carbides (cementite) [57,58]. During the deposition process, the first layer undergoes rapid cooling (quenching) due to steep temperature gradients close to the interface on top of the substrate resulting in a very fine martensite structure with some retained austenite. However, the first layer experiences phase transformation subject to reheating (tempering) phenomenon caused by subsequent layers [59]. It is known that the transgranular quasi-cleavage fracture mode is associated with the tempered martensite embrittlement (TME) mechanism, which occurs at the tempering temperature, ranging from 200 to 380 °C, in low/medium carbon and alloy steels due to the transformation of retained austenite at lath martensite boundaries [60,61]. This interlath austenite transforms to coarse cementite crystals, and cementite nucleates on the martensite boundaries, which induces large lattice distortions and a high density of dislocations that can act as crack nucleation sites and, therefore, reduces impact toughness and ductility [62,63]. 

#### 3.2.2. Phase Formation

**Figure 6:**
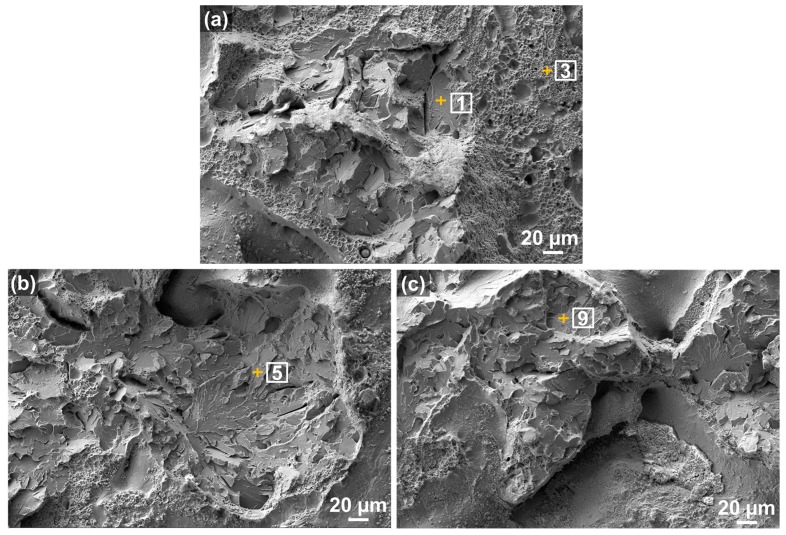
(**a**–**c**) SEM micrographs of the fracture surface (transgranular cleavage areas) for the EDS spot analyses.

EDS spot analyses were further performed on the transgranular cleavage areas, as shown in Figure 6a–c, to confirm iron carbide phase formation quantitatively as discussed in the previous section. As seen in Table 2, the wt % of C was evidently higher in the transgranular cleavage areas than the nominal composition of AISI 4140, which was confirmed as a cementite phase. Other researchers also found a similar C composition (5.72 in wt % or 22.16 in at %) and identified as cementite in similar alloy steels [64]. Darwish et al. [58] observed that cementite contained a high concentration of carbon peak values ranging from 10 to 24 at % in quasi-cleavage fracture of alloy steels. The current study also confirmed in Table 3 that C concentration ranged from 16 to 21 at %, indicating a high enrichment of carbon and carbide formation in the corresponding areas marked in Figure 6.

It was reported that the precipitation of carbide was mainly attributed to the tempering effect caused by the repeated thermal cycles inherent in the laser additive process [2], which facilities diffusion of carbon atoms and the formation of carbide in the laser-deposited part [64].

Owing to greater thermal gradients close to the interface, the lower area was restrained from precipitation and phase transformation in comparison with the intermediate and higher areas since the heat from the melt pool in the first layer on top of the substrate surface was dissipated by the substrate as a heat sink. Thus, the martensite phase is predominant near the interface as a result of the non-equilibrium phase transformation from austenite to martensite due to the rapid cooling rate. This diffusionless transformation of austenite arises only when the cooling rate is high enough to avoid diffusion of carbon atoms [28,45,65]. However, the cooling rate becomes lower as the number of newly-added layers increases and the heat inside the part is continuously accumulated, which subsequently causes the tempered martensite on the preceding layers [2,28,47]. This repeated tempering effect transforms the martensite into ferrite and precipitated carbide within the tempering temperature range above 400 °C [2,28,45,47,66]. It was observed that the precipitation of fine spheroidal cementite was dispersed along the grain boundaries of the tempered martensite matrix [45]. 

From the EDS composition analyses, a highly-enriched carbon content was detected on the fracture surface, indicating that the carbide precipitation was the main reason behind the brittle fracture mode [58]. Thus, the quasi-cleavage fracture and transgranular cracking are attributed to carbide precipitation in highly carbon enriched regions.

#### 3.2.3. Oxide Formation

**Figure 7:**
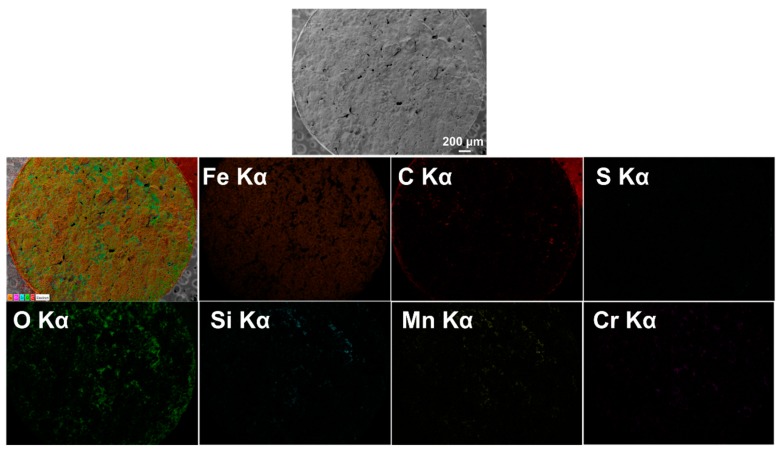
EDS element mapping on the fracture surface of specimen #3 from Figure 4.

**Figure 8:**
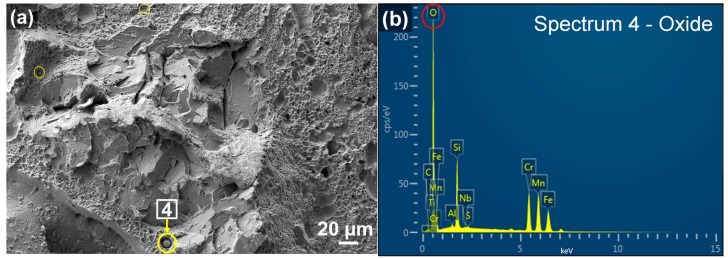
(**a**) SEM micrograph showing oxide inclusions marked by circles; and (**b**) the EDS spot spectrum of the inclusion marked by an arrow from (**a**).

**Figure 9:**
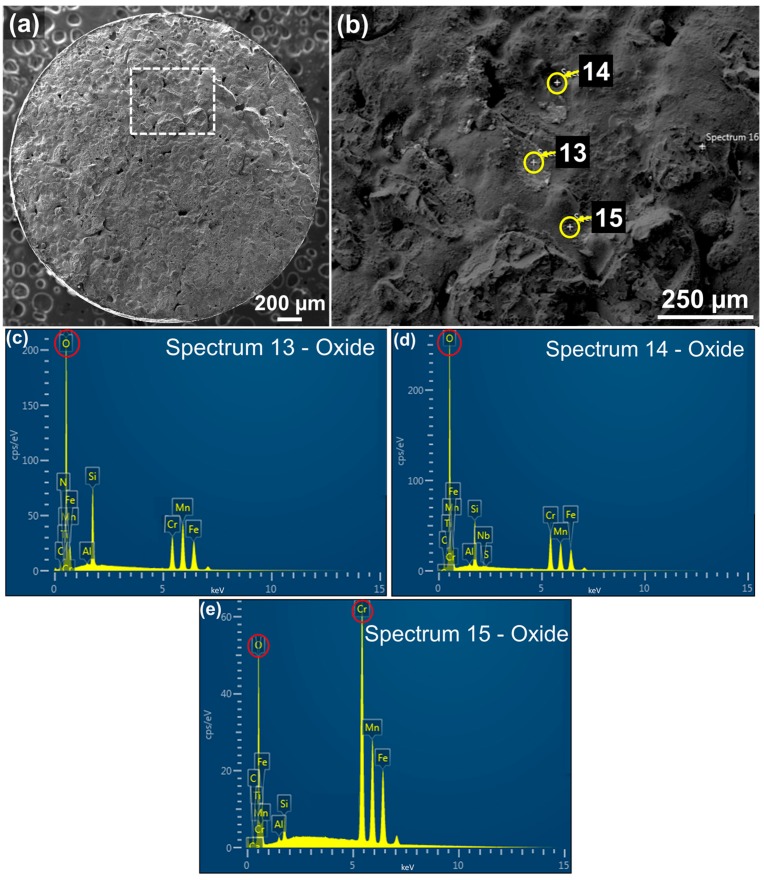
SEM micrographs showing oxide inclusions (**a**) overall fracture surface; (**b**) high magnification of a selected area from (**a**) marked by a dashed box showing oxide inclusions; and (**c**–**e**) EDS spot spectra detected on the corresponding spots marked by circles in (**b**).

Figure 7 shows EDS element mapping on the entire fracture surface, illustrating that Fe was the primary elemental constituent since it accounted for more than 96% of AISI 4140 (please refer to Table 2). However, it was observed that evident presence of O was largely distributed throughout the fracture surface along with Si, Mn, and Cr. It was considered that oxide inclusions were formed during the deposition process. To detect the elemental composition of the inclusions identified on the fracture surface, EDS spot analyses were conducted on the selected areas as shown in Figure 8 and Figure 9, respectively. As mentioned in Section 3.2.1, spherical oxide inclusions were observed in the dimple fracture area, as indicated by circles in Figure 8a. Figure 8b shows EDS spot spectrum detected on the inclusion marked by an arrow in Figure 8a, indicating that the highest peak of O, followed by Si, Mn, and Cr. The quantitative chemical composition on this inclusion was also obtained in Table 4 (in wt %) and Table 5 (in at %), which gives further evidence of higher O, Si, Mn, and Cr concentrations compared to the nominal compositions. 

Figure 9b shows the high magnification of another selected area marked by a dashed box from the entire fracture surface in Figure 9a. Spherical oxide inclusions whose morphology was similar to that of the oxide from Figure 8a were also observed as marked by circles. Figure 9c–e show EDS spot spectra detected on the inclusions marked by arrows in Figure 9b, indicating that the highest peak of O, followed by Si, Mn, and Cr. The quantitative chemical composition on this inclusion was also obtained in Tables 4 (in wt %) and 5 (in at %), which gives further evidence of the higher concentration of O, Si, Mn, and Cr contents compared to the nominal compositions.

Therefore, EDS element mapping, spot spectra, and quantitative elemental composition data all together substantiated that these oxide particles were primarily comprised of O, Si, Mn, and Cr, which implies that the oxide inclusions consist of SiO_2_, MnO, and Cr_2_O_3_ as a consequence of the chemical reactions with oxygen molecules [67]. Other researchers also reported the presence of the oxide inclusions containing SiO_2_ and MnO in different alloy steels [34,68,69,70].

There were difficulties determining stoichiometric compositions for the oxides from Table 5 since the results of the X-ray spectra were inevitably influenced by other phases in the matrix beneath the oxides at the same time with the increase of the excited volume induced by a high acceleration voltage. However, formation of the oxides can be understood using thermodynamic data. From Table 5, the highly-concentrated elements of the inclusions are Si, Mn, and Cr. According to the Ellingham diagram [71,72], these elements have a strong tendency to become oxidized by forming SiO_2_, MnO, and Cr_2_O_3_, respectively. Based on the thermodynamic data of Gibbs free energy, the oxides at the melting point of AISI 4140 (1416 °C) are formed in the following order: SiO_2_ > MnO > Cr_2_O_3_. In other words, the Gibbs free energy of SiO_2_ is the lowest and most stable and, thus, the oxidation of Si produces SiO_2_ rapidly, followed by the oxidation of Mn and Cr (i.e., MnO and Cr_2_O_3_, respectively). From a thermodynamic perspective, SiO_2_ is the most plausible oxide to be formed in AISI 4140, followed by MnO and Cr_2_O_3_. Hence, it can be inferred that the detected oxide particles would be considered SiO_2_, MnO, and Cr_2_O_3_ with evidence of the EDS data.

The existence of the oxides in the fracture surface can be described by different thermophysical properties between the oxides and AISI 4140. The melting point of AISI 4140 is 1416 °C, and that of the oxides, such as SiO_2,_ MnO, and Cr_2_O_3_ are 1600, 1945, and 2435 °C, respectively, which made the oxides difficult to dissolve during the process. As a result, the oxides remained and are trapped in succeeding layers. Moreover, the densities of SiO_2,_ MnO, and Cr_2_O_3_ (2.65, 5.43, and 5.22 g/cm^3^, respectively) are lighter than that of AISI 4140 (7.85 g/cm^3^). Consequently, due to the difference in density, the oxides would float in the molten pool, which could decrease the interlayer bonding strength [45,70].

During the high-temperature deposition process, oxygen is diffused into grain boundaries, micropores, and microvoids inside the deposited part in which it reacts to form oxides, which is called thermally-grown oxide [67]. Irrespective of the type of oxides, compressive stress is established in this thermally-grown oxide because of the diffusion. This leads to plastic deformation inside the oxide and along the interface between the oxide and the matrix in an effort to release the stress, which incurs intergranular voids attributable to the grain boundary sliding [45,73], resulting in the crack initiation and propagation at areas of stress concentration [73,74,75].

As seen on the fracture surfaces, the oxide particle inclusions degrade interlayer bonding strength. The interfaces between the oxide particles and the matrix exhibited a lack of bonding by forming voids and cracks along the interface, which could facilitate the premature fracture failure at the low stress loading and eventually have an adverse effect on the mechanical behaviors of the as-built part. Since the oxides can be regarded as crack initiators, it is necessary to minimize their inclusions in the as-fabricated part. Therefore, further study is needed to lessen the damaging effects and enhance interlayer bonding during the LENS process of AISI 4140.

## 4. Conclusions

In this study, AISI 4140 powder was deposited on AISI 4140 substrates using LENS to investigate the compatibility of LENS-deposited AISI 4140 on the corresponding wrought material by focusing on interface bond and fracture behavior of the hybrid specimens. The major findings that were obtained are:

(1) The interface between the as-deposited part and the corresponding substrate counterpart exhibits good metallurgical bonding.
(2) Through the tensile tests, all the specimens fractured within the laser-deposited region instead of the interface or the substrate, indicating that the interlayer bonding is weaker than the interface bonding.
(3) From the fractography analysis, the main causes of the fracture failure in the as-deposited part are lack-of-fusion defects, carbide precipitation, and oxide particles inclusions.
(4) The fracture failure mechanism is associated with all these factors, which deteriorates the mechanical properties and structural integrity, and causes premature failure of critical components during service.

## Figures and Tables

**Table 1:** Tensile properties comparison between hybrid samples and the wrought counterpart.

Material	UTS (MPa)	YS (MPa)	Elongation (%)
Hybrid samples in this paper *	360 ± 170	235 ± 75	2.3 ± 1.5
AISI 4140 wrought [32]	720	655	4

* Tensile fractures occurred in the as-deposited AISI 4140 zone.

**Table 2:** Distribution of elemental composition in the corresponding areas indicated in Figure 6a–c (in wt %).

Spot	C	Si	Mn	S	P	Cr	Mo	Fe	Total
1	5.30	0.31	1.12	0.00	0.00	1.32	0.31	91.65	100.00
3	5.30	0.10	0.21	0.00	0.00	0.42	0.42	93.56	100.00
5	5.06	0.10	0.62	0.10	0.00	1.24	0.00	92.88	100.00
9	4.06	0.10	0.71	0.00	0.00	1.12	0.00	94.02	100.00
Nominal	0.44	0.35	1.00	0.04	0.03	1.10	0.25	96.78	100.00

**Table 3:** Distribution of elemental composition in the corresponding areas indicated in Figure 6a–c (in at %).

Spot	C	Si	Mn	S	P	Cr	Mo	Fe	Total
1	20.55	0.40	1.01	0.00	0.00	1.21	0.20	76.62	100.00
3	20.78	0.11	0.21	0.00	0.00	0.32	0.21	78.37	100.00
5	19.89	0.21	0.53	0.11	0.00	1.06	0.00	78.20	100.00
9	16.28	0.20	0.61	0.00	0.00	1.01	0.00	81.90	100.00

**Table 4:** Elemental composition distribution of the oxide particles from EDS analyses on the corresponding spots marked in Figure 8b and Figure 9b (wt %).

Spot	O	C	Si	Mn	S	P	Cr	Mo	Fe	Total
4	28.50	5.07	7.51	22.92	0.20	0.00	19.68	0.00	16.13	100.00
13	28.10	2.56	7.79	26.46	0.00	0.00	14.97	0.00	20.10	100.00
14	31.96	4.13	5.75	18.15	0.10	0.00	21.07	0.00	18.85	100.00
15	8.90	1.82	1.11	20.02	0.00	0.00	45.50	0.00	22.65	100.00
Nominal	-	0.44	0.35	1.00	0.04	0.03	1.10	0.25	96.78	100.00

**Table 5:** Elemental composition distribution of the oxide particles from EDS analyses on the corresponding spots marked in Figure 8b and Figure 9b (at %).

Spot	O	C	Si	Mn	S	P	Cr	Mo	Fe	Total
4	50.10	11.82	7.58	11.72	0.10	0.00	10.61	0.00	8.08	100.00
13	52.10	6.29	8.18	14.26	0.00	0.00	8.49	0.00	10.69	100.00
14	55.23	9.46	5.63	9.15	0.00	0.00	11.17	0.00	9.36	100.00
15	23.26	6.47	1.62	15.17	0.00	0.00	36.60	0.00	16.89	100.00
